# Rectal mixed adenoneuroendocrine carcinoma: Case report

**DOI:** 10.1097/MD.0000000000027348

**Published:** 2021-10-08

**Authors:** Diogo J. Silva, Joana dos Santos, Ana Paula Vaz, Alexandra Mesquita

**Affiliations:** a Medical Oncology Department, Local Health Unity Matosinhos – Hospital Pedro Hispano, Porto, Portugal; b Pathology Department, Local Health Unity Matosinhos – Hospital Pedro Hispano, Porto, Portugal; c Pneumology Department, Local Health Unity Matosinhos – Hospital Pedro Hispano, Porto, Portugal.

**Keywords:** case report, mixed adenoneuroendocrine carcinoma, mixed neuroendocrine neoplasms, rectum

## Abstract

**Rationale:**

Colorectal mixed neuroendocrine–nonneuroendocrine neoplasms constitute a rare group of gastrointestinal tumors composed by both neuroendocrine and nonneuroendocrine components. Nondiagnostic macroscopic features, specific histological features, and poor awareness of the disease are responsible for the underestimated incidence and conflicting data available. Due to lack of randomized clinical trials and validated clinical guidelines, diagnostic and therapeutic approach are based on the standard of care for pure colorectal neuroendocrine carcinomas or adenocarcinomas.

**Patient concerns:**

A 76-year-old caucasian male, without relevant medical or familial history, presented a positive faecal occult blood test during colorectal cancer screening.

**Diagnosis:**

Total colonoscopy identified a rectal lesion with biopsy showing a moderate rectal adenocarcinoma staged as cT2N0M0.

**Interventions:**

Anterior resection of the rectum with right ileostomy followed by local radiotherapy with radio-sensitising chemotherapy and adjuvant chemotherapy with capecitabine 1000 mg bid plus oxaliplatin 130 mg/m^2^. Due to chronic nodular pulmonary aspergillosis and chemotherapy induced immunosuppression patient was on 400 mg/daily of oral voriconazole.

**Outcomes:**

Overall survival of 15 months after progression under first line treatment and under palliative chemotherapy with platinum plus etoposide regimen.

**Lessons:**

The reported case illustrates the challenge associated to the management of mixed neuroendocrine–nonneuroendocrine carcinomas due to lack of validated guidelines and scientific evidence. From diagnosis and staging to treatment, all steps must be tailored to individual clinical and histological features.

## 1 Introduction

Epithelial neoplasms containing 2 different cellular components, neuroendocrine and nonneuroendocrine, are a rare entity transversal to different anatomic sites. The advent of immunohistochemistry allowed for wider diagnosis, generating the need for a more accurate definition, therapeutic work-up, and prognosis.^[1]^ Despite being present nearly in every organ, better characterization was done for the subgroup arising from the digestive system. Since 2010, the World Health Organization (WHO) classification of Digestive System tumors established that mixed neuroendocrine–nonneuroendocrine neoplasms were composed at least by 30% of each component and identified as “Mixed Adenoneuroendocrine Carcinomas (MANEC).”^[2]^ Regarding the various degrees of cellular differentiation and nonneuroendocrine components pending on site of origin, “MANEC” terminology was matter of debate until 2019. The new 2019 WHO classification provided a new framework based on degree of cellular differentiation creating a generalist group of mixed neuroendocrine–nonneuroendocrine neoplasms (MiNEN).^[3]^ Re-classification as MiNEN allowed a better comprehension of the heterogeneity associated to this group of tumors, which have a spectrum of possible combinations between both components. Knowledge concerning epidemiological, clinical, and prognostic characteristics are mainly provided by single-center retrospective studies, case reports, and few systematic reviews, which limits the development of effective diagnostic and therapeutic workflows.^[1,4,5]^ MiNEC are a subgroup of gastroenteropancreatic neuroendocrine tumors (GEP-NET), which represent the second most common digestive cancers in terms of prevalence. Gastroenteropancreatic neuroendocrine tumors can arise from any site of digestive tract, being the small intestine (30.8%), rectum (26.3%), and colon (17.6%) the most common primary sites.^[1,6]^ From all colorectal tumors, MiNEN are an uncommon with low prevalence (3.2%) and commonly diagnosed in advanced stages.^[4]^

To increase the pool of clinical evidence regarding this rare entity, we describe a case of a male diagnosed with rectal MiNEN that underwent surgery and adjuvant treatment, progressing rapidly to a more aggressive disease and whose treatment was a challenge not only by the few guidelines available but also due to a rare complication.

## 2 Case report

**Figure 1:**
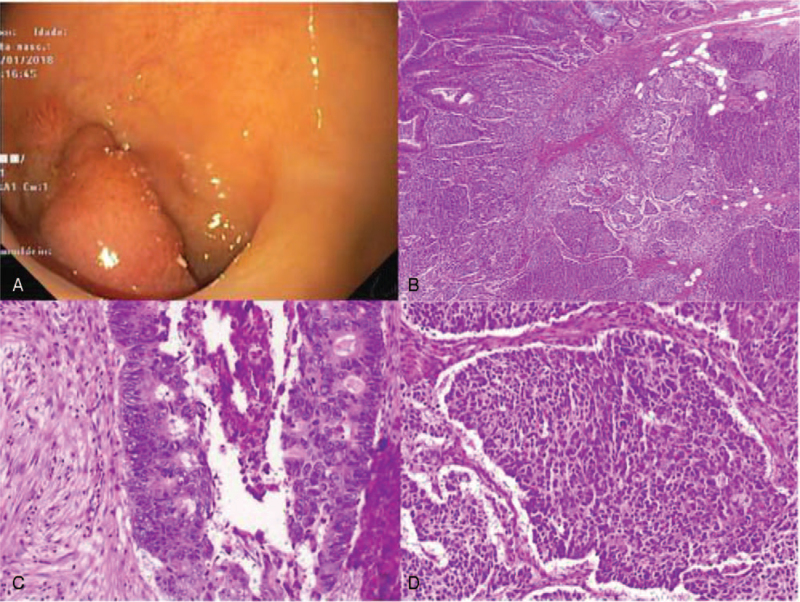
(A) Flexible proctosigmoidoscopy showing an endoluminal rectal tumor; (B) mixed adenoneuroendocrine carcinoma (H&E, 40×); (B) adenocarcinoma component composed by well-differentiated glands with marked cytological atypia (H&E, 200×); (C) small cell neuroendocrine carcinoma component, with small cells with severe atypia, scant cytoplasm, and inconspicous nucleoli (H&E, 200×).

**Figure 2:**
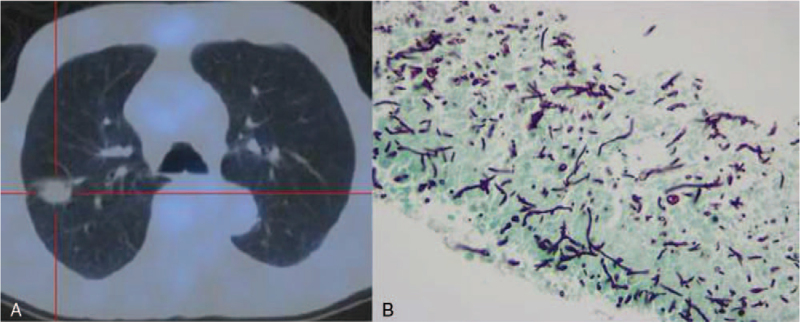
(A) Aspergilloma on the right lung upper lobe evaluated by ^18^FDG PET Scan; (B) hyphae with frequent septation, compatible with Aspergillus spp. (Grocott stain, 200×).

**Figure 3:**
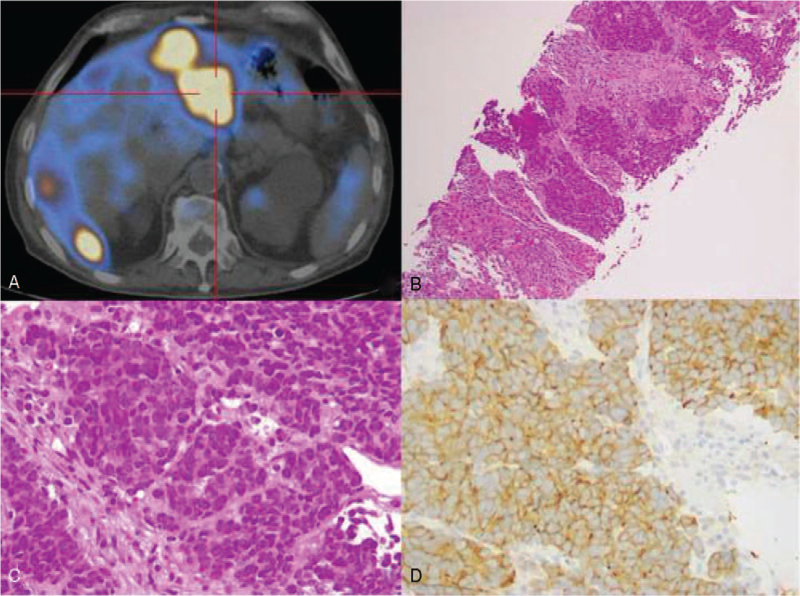
(A) High metabolic uptake of liver lesions on ^18^FDG PET-scan; (B) liver biopsy with metastatic small cell neuroendocrine carcinoma component (HE, 40×); (C) solid neuroendocrine carcinoma component with small cells, scant cytoplasm, and frequent mitotic figures (HE, 400×); (D) immunoreactivity for synaptophysin. Note the diffuse and intense staining of the cytoplasm of small cells (400×).

An asymptomatic 76-year-old male, with no past relevant medical or familial history, was diagnosed with a moderate rectal adenocarcinoma after a positive faecal occult blood test in November of 2017. Staged as cT2N0M0, an anterior resection of the rectum with right ileostomy was performed in January of 2018. Histology showed a MiNEN composed by not-otherwise specified adenocarcinoma and high-grade small cell neuroendocrine components with lymphatic plus venous invasion and two regional lymph nodes involved by both components – pT2N1bR0 according to American Joint Committee on Cancer 8th Edition. Immunohistochemically, both components were positive for CAM 5.2; the neuroendocrine neoplastic cells were negative for chromogranin but showed positivity for synaptophysin, with a proliferative index over 50% (Fig. 1). Considering the new histological information obtained after surgery, a 68Gallium-labeled [1,4,7,10-tetraazacyclododecane–1,4,7,10-tetraacetic acid]-NaI3-octreotide positron emission tomography scan showed a discrete hypermetabolic unspecific nodule on right lung lower lobe. After multidisciplinary discussion, patient was proposed to local radiotherapy with radio-sensitising chemotherapy followed by adjuvant chemotherapy with capecitabine 1000 mg bid plus oxaliplatin 130 mg/m^2^ (CAPOX protocol) assuming that the adenocarcinoma component was dominant and after literature review. A dose reduction of oxaliplatin was done due to clinical fragile condition and age (Eastern Cooperative Oncology Group 2). Having completed local radiotherapy with radio-sensitising capecitabine and 5 cycles of adjuvant chemotherapy, Computed Tomography scan guided transthoracic biopsy of the unspecific hypermetabolic nodule showed presence of necrotic tissue with Aspergillus spp. suggestive of chronic Aspergillosis (Fig. 2). After assessment by Pneumology, patient was initiated on 400 mg/daily oral voriconazole and adjuvant chemotherapy was postponed. After 14 days of treatment, a cytocolestathic pattern was found and an abdominal ultrasound showed presence of two nodules with inconclusive sonographic characteristics. Regarding this, an assessment by both Magnetic Resonance Imaging and positron emission tomography (PET) with 2-deoxy-2-[fluorine-18]fluoro-D-glucose scan was suggestive of secondary lesions. Presence of small cell neuroendocrine carcinoma was confirmed by biopsy (Fig. 3). Facing the evidence of progression, and its aggressive neuroendocrine component, multidisciplinary Oncology group decided for palliative chemotherapy with platinum plus etoposide regimen adapted to performance status. Once Aspergillosis was considered controlled under voriconazole and liver cholestasis was solved, dose was readjusted to standard protocol. After 3 cycles, PET with 2-deoxy-2-[fluorine-18]fluoro-D-glucose scan showed a mixed metabolic response of secondary lesions and a clinical patient benefit was noticed. Therefore, palliative chemotherapy was maintained, only suspended due to obstructive jaundice with clinical deterioration needing in-hospital admission for further care. Biliary calculi were removed by endoscopic retrograde cholangiopancreatography and a stent placed. Despite jaundice resolution, clinically deterioration became more evident during inward stay. 18F-FDG PET scan documented disease progression with new metastases on left lung upper lobe and increased metabolic activity of liver disease. Regarding this, patient was referred to the Palliative Care Team for symptomatic control measures, having an overall survival of 15 months.

## 3 Discussion

Neuroendocrine neoplasms are a rare and heterogenous group of tumors arising from multiple anatomic sites with characteristic histology and immunohistochemistry.^[7]^ MiNEN express a wide spectrum of biomarkers of neuroendocrine differentiation (Chromogranin A, CD56, and synaptophysin) on the neuroendocrine compartment as well as site specific markers such as CK20 and Caudal Type Homeobox 2 on adenocarcinoma compartment. Its presence along different organs led to elaboration of various site-specific terminologies and definition criteria, making an objective and transversal communication between pathologists, researchers, and physicians hard to achieve.^[8]^ In order to tackle this point, the International Agency for Research on Cancer and WHO elaborated an expert consensus in 2017, proposing that under the broader definition of neuroendocrine neoplasm was defined a group of well differentiated tumors whose potential to metastasize or invade depend on site and type (NET), together with a group of aggressive, poorly differentiated and high-grade tumors (neuroendocrine carcinomas: NEC).^[9]^ The need for two different classification frameworks urged from the data suggesting that well-differentiated NET and poorly differentiated NEC are not biologically related, having different degrees of biological aggressiveness, risk factors, responses to therapy, and relation to nonneuroendocrine neoplasms. Moreover, in some anatomic sites (i.e., lung, digestive system) tumors exhibit both nonneuroendocrine and neuroendocrine components in a separated or intimately related form with a distinct biologic behavior from NET or NEC.^[8]^ Although recognized as an important subgroup of NENs, not until the new 2019 WHO classification for Digestive Tumors was “mixed neoplasm” formally part of the classification framework. This new classification acknowledged the relevant differences between NET and NEC, validating the concept of “mixed neuroendocrine–nonneuroendocrine neoplasms.” ^[3]^ Pathophysiology of MiNEN remains unclear, with 3 main theories trying to explain how this group of neoplasm originate. The first one postulates that MiNEN originate from 2 different precursors that arise in the same location; the second theorises a monoclonal origin where both components arise from a pluripotent epithelial stem cell capable of bidirectional differentiation; the third agrees with a monoclonal origin but hypothesises a neuroendocrine differentiation from the nonneuroendocrine component through successive molecular or genetic changes.^[1,10]^ All try to justify the histological features and patterns found on MiNEN, with some tumors presenting both neuroendocrine and nonneuroendocrine components mingled together (composite tumors); others showing 2 different cellular masses divided by a transition zone (collision tumors) or a unique cellular population of mixed neuroendocrine–nonneuroendocrine components at cellular level (amphicrine tumors).^[8,10]^ According to the WHO classification of 2019, the threshold of each one of the components must be bigger than 30%, being the only rational for this definition the fact that an inferior relation would not be biological significant. As stated, MiNEN can be formed by a wide range of cellular combinations. One of those “subtypes” is the “MANEC,” a rare tumor normally associated with biologic aggressiveness and poor outcome.^[4,11]^ Despite formally acknowledged by the WHO, International Agency for Research on Cancer and European Neuroendocrine Tumour Society, epidemiological, clinical, and prognostic data on MiNEN and MANEC are still insufficient. Frizziero et al wrote a systematic review summarizing the best evidence available, stating that the most common sites of origin were appendix (60.3%), colon-rectum (14.5%), and stomach (6.7%); males were more frequently diagnosed (65.6%) in general and site specific. Stage at diagnosis was also assessed, being localised disease more common (81.6%) than advanced or metastatic disease (18.4%). Histological analysis described a nonneuroendocrine component in 29.9%, adenocarcinoma in 92.2%, or squamous cell carcinoma in 2.5%. To better understand the behavior of the case reported, we should also focus on the existing evidence on gastrointestinal mixed adenocarcinomas. Wang et al performed a retrospective analysis of Gastrointestinal MANECs registered in the Surveillance, Epidemiology, and End Results Program from the Cancer National Institute between 2000 and 2016. With a median age at diagnosis of 59 years old (interquartile range: 50–67 years), the majority were Caucasian (81.2%) men (53.8%) with regional (40.4%) or localized disease (27.0%). Bearing this in mind, the case reported corroborates findings of Frizziero et al and Wang et al, being a Caucasian male diagnosed with a localized rectal mixed adenoneuroendocrine carcinoma.

Concerning diagnosis, colonoscopy is the most reliable mean of diagnosis for colorectal MiNEN. Endoscopic findings are morphologically similar to colon adenocarcinoma, presenting as a semi-circular tumor with deep ulceration or as prominent mass occupying the lumen.^[12]^ Not having macroscopic diagnostic alterations, histology, and immunohistochemistry are crucial for establishing a correct diagnosis. However, sometimes biopsy obtained specimens do not obtain the full spectrum of histological patterns. MiNEN usually present as a high malignant composite neoplasm formed by an adenomatous (villous/tubule-villous) or carcinomatous (adenocarcinoma/squamous cell carcinoma) component with a poorly differentiated neuroendocrine carcinoma (small or large cells).^[1,10,13]^ Translating to the case reported, this technical difficulty explains the first diagnosis as rectal adenocarcinoma on biopsy and the subsequent reformulation to MiNEN when full specimen was obtained.

In 2012, La Rosa et al proposed a prognostic classification based on grade of differentiation of neuroendocrine component, setting the basis for the redefinition of Gastrointestinal MiNEN classification framework both in 2017 and later in 2019. Subdividing Gastrointestinal MANEC into high-grade malignant (NEC) and intermediate grade malignant (G1/G2 and amphicrine carcinoma); introducing the new term “Mixed Adenoneuroendocrine Tumor,” the proposal framework stated that degree of differentiation would influence prognostic. Additionally, neuroendocrine cellular subtype could also convey prognostic features with studies showing better outcomes for large cell subtype. Other relevant point during histological analysis is immunohistochemistry for chromogranin A, synaptophysin, and/or CD56. According to the WHO classification of 2019, neuroendocrine differentiation is confirmed by positivity for synaptophysin (being reported as the most sensitive neuroendocrine marker) or chromogranin A (the most specific marker). Despite being formally recognized as a clinical entity by the WHO and European Neuroendocrine Tumour Society, no specific validated treatment guidelines are available. National Comprehensive Cancer Network Guidelines for Neuroendocrine and Adrenal Tumors (V1.2019) advise that gastrointestinal tumors with mixed histology should be managed and treated according to colon cancer guidelines.^[14]^ National Comprehensive Cancer Network, ENTS, and North America Neuroendocrine Tumour Society advise surgical resection (low anterior or abdominoperineal resection) for neuroendocrine neoplasm > 2 cm with invasion of muscularis propria, lymphovascular, or locoregional lymph nodes.^[14,15]^ For metastatic disease, resection is advised if complete resection is possible. Regarding adjuvant therapy, multimodal approach with radiotherapy plus chemotherapy is advised even if R0 resection is achieved as MANEC have a high recurrence rate. Regimens based on platinum plus etoposide/topotecan, FOLFOX, or carboplatin plus etoposide have been reported in this setting.^[12,15]^

For recurrent or unresectable disease, selection of chemotherapy regimen is complex and should be done after confirmation of which component is responsible for the metastatic disease. A non-invasive option to access the presence of neuroendocrine component is somatostatin receptor scintigraphy.^[12]^ On one hand, if metastatic disease is mainly adenocarcinoma, a 5-fluorouracil-based regimen plus oxaliplatin should be used as first line. On the other hand, when NEC is the main component, a cisplatin plus etoposide regimen seems more appropriated. Some regimens of cisplatin plus irinotecan or paclitaxel, carboplatin, and etoposide have been reported with good outcomes.^[12,15]^

Our case comprises an adequate example of how tailored a therapeutic approach needs to be when dealing with MiNEN. Multidisciplinary team decided for treatment using colon-rectal cancer adenocarcinoma protocols, adjuvant radio-chemotherapy for local control, and adjuvant chemotherapy based on platins and fluoropyrimidines, according to most of literature reviews and guidelines. Progression during adjuvant regimen led to a protocol reframe to carboplatin plus etoposide chemotherapy, once neuroendocrine small cell component was found in the metastatic disease. As stated before, MiNEN have an aggressive biological behavior usually with poor outcomes. However, data on outcomes and prognostic factors is limited. For localized disease, median overall survival ranges between 14 and 75 months; in advanced setting, median overall survival ranges from 10 to 18 months. Analysis including non-specified stage, localized, and advanced disease show median overall survival between 10.5 and 78 months.^[5]^ When comparing survival outcomes with other gastrointestinal tumors, MiNEN have a worse survival outcome than carcinoid tumors but better than adenocarcinoma. Despite not having validated prognostic factors, some factors have been proposed as predictors of poor prognosis: diagnosis after 60 years old; regional and distant disease; poorly or undifferentiated tumor's grade; positive lymph nodes; and tumor size > 2 cm.^[5]^ In this case report, the patient had all these poor prognostic factors. The diagnosis of a pulmonary Aspergillosis was also a factor of concern, making it difficult to stratify the best treatment option with the need of protocol adaptations.

## 4 Conclusion

We report a case of a rare rectal mixed adenoneuroendocrine carcinoma with an overall survival of 15 months after surgical resection and progression under adjuvant treatment. Management of this group of tumors is challenging due to lack of validated guidelines and scientific evidence. From diagnosis and staging to treatment, all steps must be discussed in multidisciplinary teams and tailored to individual clinical and histological features. Regarding its rarity, multicentric and translational studies are difficult to be implemented but needed to increase the current knowledge.

## Author contributions

AM was the Medical Oncologist responsible for chemotherapy treatment, data assessment, and manuscript review. DJS was responsible for literature review, data assessment, manuscript writing, and reviewing. JS was responsible for histology assessment, imaging, and manuscript review; APV was the Pneumologist responsible for Aspergillosis management, treatment, and manuscript review.

**Conceptualization:** Diogo Silva, Alexandra Mesquita.

**Data curation:** Diogo Silva, Joana dos Santos, Alexandra Mesquita.

**Formal analysis:** Diogo Silva, Alexandra Mesquita.

**Methodology:** Diogo Silva.

**Supervision:** Alexandra Mesquita.

**Writing – original draft:** Diogo Silva.

**Writing – review & editing:** Ana Paula Vaz, Alexandra Mesquita.
